# Management, treatment and prognostic significance of lateral lymph node metastases in rectal cancer—a regional cohort study

**DOI:** 10.1007/s00384-021-04018-1

**Published:** 2021-09-06

**Authors:** E. Agger, V. Åkerlund, O. Ekberg, F. Jörgren, M. L. Lydrup, P. Buchwald

**Affiliations:** 1 Department of Surgery, Skåne University Hospital, Lund University, Malmö, Sweden; 2 Department of Surgery, Västmanland Hospital, Västerås, Sweden; 3 Department of Radiology, Skåne University Hospital, Lund University, Malmö, Sweden; 4 Department of Surgery, Helsingborg Hospital, Lund University, Helsingborg, Sweden

**Keywords:** Rectal cancer, Lateral lymph node metastases, Chemoradiotherapy

## Abstract

**Purpose:**

Lateral lymph node metastases in rectal cancer remain a clinical challenge. Different treatment regimens have been suggested. This retrospective regional cohort study examines outcome after combined oncological and surgical treatment of MRI-positive lateral lymph nodes (LLN).

**Methods:**

Data from the Swedish Colorectal Cancer Registry (SCRCR) and patient records were used for retrospective analysis of resected high-risk rectal cancers between 2009 and 2014. The aim was to compare tumour characteristics, neoadjuvant therapy, recurrence and outcome after lateral lymph node dissection.

**Results:**

One thousand and one hundred nineteen cases were identified and after exclusion 344 patients with cT3–T4 ≤ 10 cm from the anal verge were analysed. Thirty (8.7%) patients with MRI-positive LLN were identified. Synchronous distant metastases were associated with MRI-positive LLN (*p*-value 0.019). Long-course chemoradiotherapy was clinical practice in cases of MRI-positive LLN. No differences in local (*p*-value 0.154) or distant (*p*-value 0.343) recurrence rates between MRI-positive LLN patients and MRI-negative patients were detected. Only four patients underwent lateral lymph node dissection (LLND). There was no significant difference in overall survival during follow-up between the MRI-negative (CI at 95%; 99–109 months) and MRI-positive group (CI at 95%; 69–108 months; *p*-value 0.14).

**Conclusion:**

Lateral lymph node metastases present a challenging clinical situation. The present study shows that combination of neoadjuvant therapy and selective LLND is an applicable strategy in cases of MRI-positive LLN.

**Supplementary Information:**

The online version contains supplementary material available at 10.1007/s00384-021-04018-1.

## Introduction

Lateral lymph node (LLN) stations are located outside the normal reign of standard total mesorectal excision (TME) in rectal cancer surgery. Lymph node metastases outside the mesorectum might be located along the iliac vessels in the pelvis, in the inguinal fossa and in paraaortic lymph node stations. Metastases along the iliac vessels are considered N2-disease whereas tumour growth in the inguinal or paraaortic lymph nodes is considered M1 disease [1].

Tumours located in the lower two-thirds of the rectum, advanced T-stage and histopathological risk factors are associated with increased risk of lateral lymph node metastases (LLNM) [2]. LLNM are associated with an increased risk of local recurrence (LR) and decreased survival [2]. Pelvic MRI is the most accurate method to identify LLNM before and after neoadjuvant therapy [3].

Management of patients with LLNM differs between the East and the West with Japanese guidelines recommending LLN dissection (LLND) as primary approach whereas in the West, patients with LLNM are often treated with long-course chemoradiotherapy (CRT) according to neoadjuvant protocol used in locally advanced rectal cancer followed by standard TME [4–6]. Studies by Akiyoshi et al. and Ogura et al. suggest that neoadjuvant therapy might not be sufficient to reduce the risk of LR in patients with persisting MRI-positive LLN at re-evaluation [7, 8]. Persisting LLN enlargement has been shown to correlate with risk of LR after long-course CRT [5].

Some authors argue that LLND should be performed in all patients with MRI-positive LLN, a strategy that might decrease the risk of LR and increase disease-free survival [9]. Current Swedish guidelines mandate that MRI-positive LLN should be treated as locally advanced rectal cancer with long-course CRT [10]. Persisting MRI-positive LLN with size > 6 mm or other high-risk features after neoadjuvant therapy may be considered for excision, primarily to reduce risk of LR [5, 10].

This study aims to describe results and practises in a regional high-risk rectal cancer cohort treated with neoadjuvant therapy and TME-surgery according to current Swedish guidelines [10].

## Methods

Patients treated in southern Sweden (Skåne) for rectal cancer with TME-surgery; anterior resection, Hartmann’s procedure or abdominoperineal resection between January 1, 2009, and December 31, 2014, were identified via the Swedish Colorectal Cancer Registry (SCRCR) [11, 12].

After identification, patients were assessed for inclusion. Patients with tumour ≤ 10 cm from the anal verge, stage cT3–4 and cN1–2, negative circumferential (CRM) and distal (DRM) resection margins and available MRI were included, followed by review of medical records. The primary MRI reports were examined, and all cases with noticeable lymph nodes in vicinity or outside the mesorectal fascia were subjected to a secondary review of the original MRI, performed by an experienced GI radiologist (OE) according to predefined criteria (Supplement 1).

Results of histopathological examination in MRI-positive LLN patients subjected to LLND were analyzed. All histopathological specimens had been examined by a dedicated GI-pathologist within the clinical routine.

### Definitions

#### Lateral lymph nodes

Lymph nodes located outside the mesorectal fascia along the iliac vessels. Inguinal and paraaortic lymph nodes were not included in the definition of LLN since they constitute M1 disease [1]. Patients with only inguinal or paraaortic lymph node metastasis were included in the control group.

#### Pathological lymph node

Malignant features of lymph nodes were defined as indistinct borders, heterogeneous signal or attenuation and round shape. Nodes with short axis of < 5 mm needed three malignant characteristics to be deemed MRI-positive, and those with short axis of 5–9 mm needed two malignant characteristics. Lymph nodes with short axis measurement of > 9 mm were always deemed MRI-positive [13]. In this study, MRI-positive LLN was equal to LLNM.

#### Rectal cancer

Rectal cancer was defined as an adenocarcinoma with the lower border located ≤ 15 cm from the anal verge measured with rigid sigmoidoscopy.

#### Distant metastasis

Distant metastasis (DM) was defined as tumor recurrence in an organ outside the small pelvis such as lungs, liver, lymph nodes outside the pelvis, peritoneum and/or any other distant organ.

#### Local recurrence

LR was defined as local extraperitoneal tumour recurrence, tumour growth in local lymphatic nodes, intraluminal tumour recurrence or peritoneal tumour growth below the promontory.

### Statistical analysis

Categorical variables were presented as number and proportions in percentages. Numerical data were reported as means with interquartile range. Pearson’s chi-square test, Fisher’s exact test or two-tailed *T*-test were used for intergroup comparisons. Survival analysis was performed with Mantel-Cox regression and presented with a Kaplan–Meier survival plot (Supplement 2). Missing data was excluded when calculating differences between groups.

Statistical analyses were conducted using IBM® SPSS® Statistics version 25.00 for Windows® (IBM Corp, Armonk, NY, USA). *P*-Value < 0.05 was considered significant.

## Results

### Study population

**Fig. 1:**
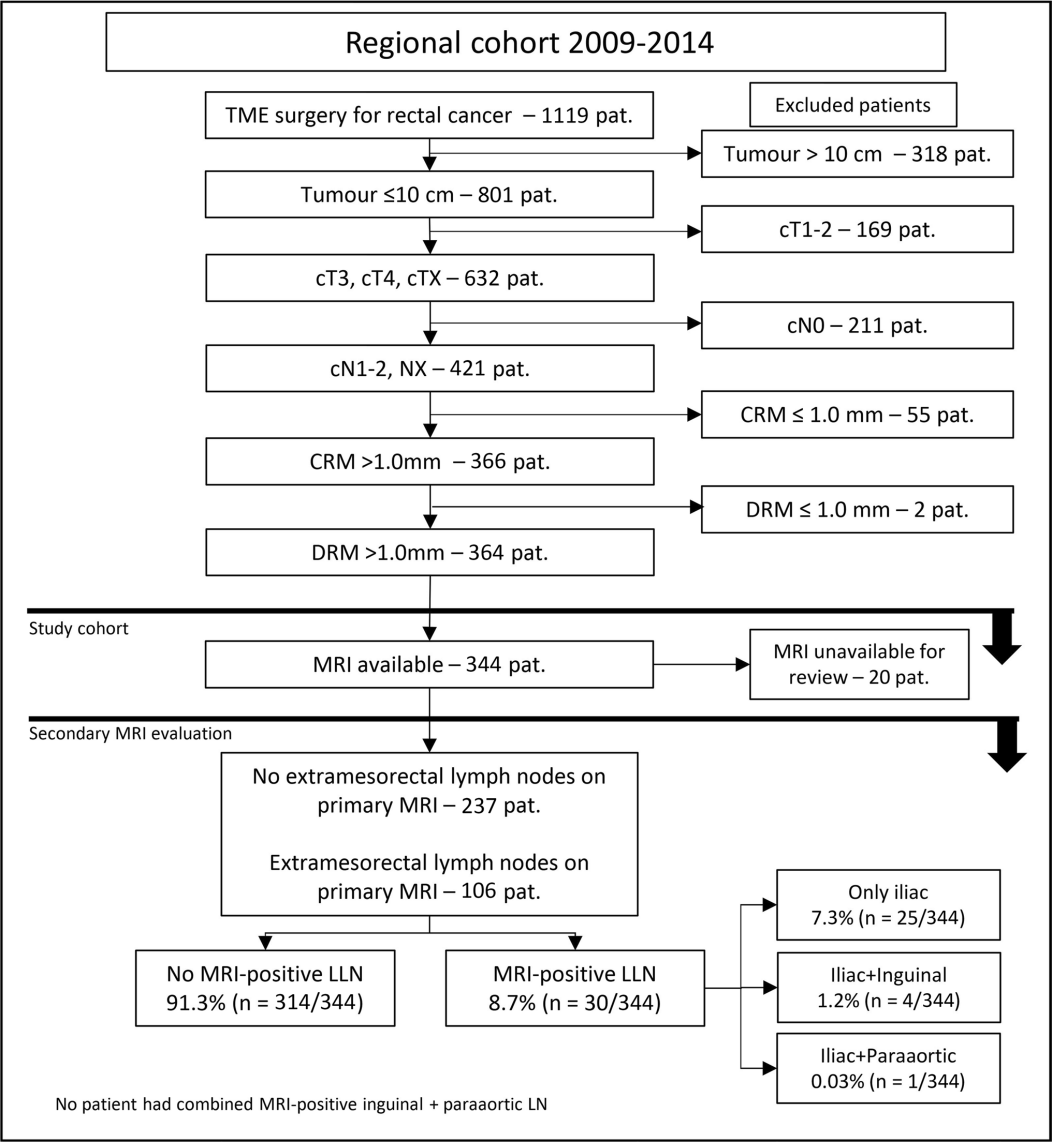
Study flow chart—CRM, circumferential resection margin; DRM, distal resection margin; LLN, lateral lymph node; LN, lymph node

A total of 1119 patients were identified from SCRCR and assessed for inclusion. Seven hundred fifty-four patients were excluded according to predefined criteria (Fig. 1). The remaining 364 patients’ medical records were reviewed which resulted in further exclusion of twenty patients for whom no radiological record was available. The study cohort of 344 patients was subjected to secondary radiological review. The mean follow-up time in the study population was 75 months (IQR 55–99) after surgery.

Thirty (8.7%) patients with MRI-positive LLN were identified. Out of these, 25 (7.3%) had only MRI-positive iliac lymph nodes, four (1.2%) patients had both iliac and inguinal positive nodes and one patient (0.03%) had a combination of iliac and paraaortic positive nodes. Four (1.2%) patients had positive inguinal nodes alone, and four (1.2%) patients had positive paraaortic nodes alone (Table 1). In patients with MRI-positive LLN only, 92% (23/25) were found at one location and 8% (2/25) at multiple locations (Table 2).

**Table 1:** Cross-tabulation of patients with MRI-positive lymph nodes in multiple locations LLN, lateral lymph node

Patients with MRI-positive LLN			
	Iliac	Inguinal	Paraaortic
Iliac	25		
Inguinal	4	4	
Paraaortic	1	0	4

**Table 2:** Locations of MRI-positive LLN. Among the majority of patients, MRI-positive LLNs were found at one location. Two patients had positive LLNs in multiple locations

Right internal iliac artery	Right external Iliac artery	Right obturator artery	Right common Iliac artery
12	2	0	4
48.0%	8.0%	0.0%	16.0%
**Left internal** **iliac artery**	**Left external** **Iliac artery**	**Left obturator** **artery**	**Left common** **Iliac artery**
9	2	3	2
36.0%	8.0%	12.0%	8.0%

## Tumour characteristics and therapy

Patients with MRI-positive LLN had a higher prevalence of synchronous distant metastasis. In the node-positive group, 12 (40.0%) patients were classified as cM1 versus 40(15.1%) patients in the node-negative group (*p*-value < 0.001). DM to locations other than inguinal and paraaortic LN-stations were frequent in both groups, 58.3% and 80%, respectively (Supplement 3). However, in patients with MRI-positive LLN and synchronous DM, 33.0% (4/12) had MRI-positive inguinal metastases compared to 10.0% (4/40) in the MRI-negative LLN group.

Almost all LLN-positive patients, 93.3% (28/30) received neoadjuvant therapy with the majority, 60.0% (18/30) treated with long-course CRT and 33.3% (10/30) with short-course radiotherapy (RT). 89.5% (281/314) of patients in the LLN-negative group received neoadjuvant therapy and, in this group, short-course RT was the most prevalent treatment accounting for 38.5% (121/314). Patients with cT4 tumours received long-course CRT in 75.0% (0–5-cm from anal verge; 59.0%, 6–10 cm from anal verge 40.9%) of cases and short-course RT in 17.0% of cases. In patients with cT4 tumours undergoing short-course RT, all but one were located within 5 cm from the anal verge. Complete pathological response occurred after short-course RT and long-course CRT without any statistically significant difference between groups (*p*-value 0.63). In 4.6% (13/281) of MRI-negative LLN and 7.1% (2/28) of MRI-positive patients, no residual tumour was found during histopathological examination.

Low tumours (0–5 cm from the anal verge) accounted for 40.4% (139/314) and medium height (6–10 cm from the anal verge) tumours 59.6% (205/344) with comparable distribution between groups. Abdominoperineal resection (APR) seemed the more favoured surgical procedure for patients with MRI-positive LLN status; 46.7% of patients in this group had low tumour but 70.0% had an APR (Table 3). In MRI-positive LLN patients with medium tumour height, seven patients had an APR, two patients a Hartmann’s resection and seven patients an anterior resection (Supplement 4).

**Table 3:** SCRT, short-course radiotherapy; LCRT, long-course chemoradiotherapy; CHT, chemotherapy; AR, anterior resection; APR, abdominoperineal resection

		All patients		MRI-negative LLN		MRI-positive LLN		*p*-value
		*n*	%	*n*	%	*n*	%	
Patients		344	100.0%	314	91.3%	30	8.7%	
Sex	Male	206	59.9%	185	58.9%	21	70.0%	0.237
	Female	138	40.1%	129	41.1%	9	30.0%	
Age	Mean	66.4		66.4		66.2		0.238
ASA	1	95	27.6%	86	27.4%	9	30.0%	0.414
	2	185	53.8%	171	54.5%	14	46.7%	
	3	57	16.6%	51	16.2%	6	20.0%	
	4	3	0.9%	2	0.6%	1	3.3%	
	*Missing*	*4*	1.2%	*4*	1.3%	*0*	0.0%	
Neoadjuvant therapy	None	26	7.6%	25	8.0%	1	3.3%	0.128
	SCRT	170	49.4%	160	51.0%	10	33.3%	
	LCRT	139	40.4%	121	38.5%	18	60.0%	
	Other	9	2.6%	8	2.5%	1	3.3%	
Surgical procedure	APR	188	54.7%	167	53.2%	21	70.0%	0.209
	Hartmann	33	9.6%	31	9.9%	2	6.7%	
	AR	123	35.8%	116	36.9%	7	23.3%	
Tumour height (cm)	Low 0–5	139	40.4%	125	39.8%	14	46.7%	0.465
	Medium 6–10	205	59.6%	189	60.2%	16	53.3%	
cT-stage	T3	227	66.0%	209	66.6%	18	60.0%	0.250
	T4	88	25.6%	77	24.5%	11	36.7%	
	TX	29	8.4%	28	8.9%	1	3.3%	
cN-stage	N1–2	235	68.3%	210	66.9%	25	83.3%	0.064
	NX	109	31.7%	105	33.4%	5	16.7%	
cM-stage	M0	292	84.9%	274	87.3%	18	60.0%	< 0.001
(Synchronous)	M1	52	15.1%	40	12.7%	12	40.0%	
pT-stage	T0–2	108	31.4%	98	31.2%	10	33.3%	0.744
	T3–4	233	67.7%	213	67.8%	20	66.7%	
	TX	3	0.9%	3	1.0%	0	0.0%	
pN-stage	N0	193	56.1%	173	55.1%	20	66.7%	0.407
	N1–2	149	43.3%	139	44.3%	10	33.3%	
	NX	2	0.6%	2	0.6%	0	0.0%	
Perineural growth	Yes	70	20.3%	67	21.3%	3	10.0%	0.135
	No	271	78.8%	244	77.7%	27	90.0%	
	*Missing*	*3*	0.9%	*3*	1.0%	*0*	0.0%	
Vascular growth	Yes	59	17.2%	55	17.5%	4	13.3%	0.547
	No	282	82.0%	256	81.5%	26	86.7%	
	*Missing*	*3*	0.9%	*3*	1.0%	*0*	0.0%	
Adjuvant therapy	None	235	68.3%	213	67.8%	22	73.3%	0.536
	CHT	109	31.7%	101	32.2%	8	26.7%	
Local recurrence	Yes	16	4.7%	13	4.1%	3	10.0%	0.154
	No	328	95.3%	301	95.9%	27	90.0%	
Distant metastasis	Yes	111	32.3%	99	31.5%	12	40.0%	0.343
(Metachronous)	No	233	67.7%	215	68.5%	18	60.0%	
Follow-up time	Mean (m)	75	IQR55–99	76	IQR60–100	65	IQR34–97	< 0.001

Histopathological risk factors, both perineural growth and lympho-vascular infiltration, were equally prevalent in both groups (Table 3). Among LLN-negative patients 21.3% and 17.5% were diagnosed with perineural growth and lympho-vascular infiltration respectively compared to 10.0% (*p*-value 0.14) and 13.3% (*p*-value 0.55) in LLN-positive patients.

Patients in both groups were treated with adjuvant chemotherapy (CHT) to a similar extent. In the MRI-negative LLN group 32.2% (101/314) received adjuvant CHT compared to 26.7% (8/30) in the MRI-positive LLN group (*p*-value 0.54).

## Outcome of lateral lymph node dissection

Review of surgical notes showed only four cases of LLND in this cohort; all patients had received neoadjuvant therapy. Histopathological examinations revealed one case of metastatic adenocarcinoma, one case of metastatic prostate cancer and in two cases benign lymph nodes. The four patients who underwent LLND were re-evaluated after neoadjuvant therapy with both MRI and FDG-PET-CT with persistent malignant LN morphology. No major complication or post-operative mortality was found among these patients.

## Recurrence and survival

In total, 16 (4.7%) LR and 111 (32.3%) DM were diagnosed. LR rate was 10.0% and 4.1% in the MRI-positive LLN and MRI-negative LLN groups, respectively (*p*-value 0.15). DM rate was 40.0% in MRI-positive LLN patients compared to 31.5% in MRI-negative LLN patients (*p*-value 0.34). There was no significant difference in overall survival during follow-up between the MRI-negative (CI at 95%; 99–109 months) and MRI-positive group (CI at 95%; 69–108 months; *p*-value 0.14). Kaplan–Meier survival plot is provided in Supplement 2.

## Discussion

Management of suspected LLNM in rectal cancer surgery is a challenging clinical situation. In this regional cohort study, we examine a treatment strategy reliant on CRT and LLND only in selected patients. Our results indicate that this was a feasible strategy with no statistically significant differences in recurrence rates between groups.

Synchronous DM, i.e., outside inguinal or paraaortic lymph nodes, was significantly more prevalent among patients with MRI-positive LLN at diagnosis, despite no significant differences in pre- or postoperative tumour stage and histopathological risk factors. Simultaneous inguinal and paraaortic lymph node metastases were uncommon and are in coherence with observations in previous studies where such metastases are found in 0–2% of rectal cancer patients [14, 15]. Perineural growth and lympho-vascular invasion are known risk factors for recurrence in rectal cancer and are found in approximately one in every four to five patients with rectal cancer during histopathological examination [16, 17]. The significance of extramural vascular invasion as a risk factor of LLNM has recently been recognised and requires further studies [18]. The specific prognostic value of these risk factors varies however with other tumour characteristics, especially in high-risk tumours [19]. This may explain why lympho-vascular invasion was similar despite presence of positive LLN. Notably, after histopathological examination, the number of positive mesorectal lymph nodes was comparable between groups. Neoadjuvant therapy is known to reduce the number of detected mesorectal lymph node metastases, and LLNM may exist without lymph node involvement in the mesorectum [20, 21].

The current study identified 8.7% MRI-positive LLN; however, the true prevalence of LLNM in rectal cancer is unknown, in studies based on histopathological examination, a prevalence of between 10 and 20% is often reported [22]. Although pelvic MRI is a highly sensitive and specific method to detect LLNM, it might underestimate the true prevalence of LLNM [23]. Since LLNM is relatively uncommon, a larger sample size might be necessary to detect significant differences in recurrence parameters.

Some studies have shown reduced risk of local recurrence when LLND is performed, both in patients with or without MRI-suspected LLNM [9, 24]. However, in a meta-analysis by Fahy et al. no such benefit was observed [25]. Moreover, it has been suggested that LLND results in longer operative time, greater intraoperative blood loss, increase in postoperative complications and increased incidence of dysuria and impaired sexual functions without subsequent improvement of survival [26–28].

The use of CRT in patients with MRI-suspected LLNM is known to decrease LR risk and increase disease-free survival, both in combination with LLND and with TME without LLND [29, 30]. In the current study, almost all patients with MRI-positive LLN received neoadjuvant therapy, but very few were subjected to LLND. Additionally, in the present study cohort, most patients with MRI-positive LLN were re-evaluated with MRI after long-course CRT. Patients who received short-course RT were principally not re-evaluated with MRI. In accordance with Swedish treatment guidelines, patients with complete regression of MRI-suspected LLNM, LLND is not mandatory and only MRI-suspected LLNM which do not respond to neoadjuvant therapy are considered for LLND. This might explain the very few LLND performed in this cohort. Furthermore, PET-CT was only used occasionally during the study period but might further improve diagnosis of residual tumour in LLN after CRT and may aid surgical decision regarding LLND [31]. Swedish guidelines do not mandate PET-CT neither in primary diagnostics nor in re-evaluation; currently, its primary role is diagnosis of extra-pelvic metastases or suspected recurrence [10].

In this cohort with high-risk tumours, more extensive surgical strategies might be expected in both groups; however, patients with MRI-positive LLN were more often treated with APR. The reason for this is unknown and was not related to corresponding abundance of low tumours in this group. In choosing surgical strategy after long-course CRT, APR was likely often the preferred option to avoid complications related to anastomosis and risk of low anterior resection syndrome.

Treatment strategies vary significantly across continents and management remains a subject for debate [5, 6]. Future studies are warranted to address novel neoadjuvant therapies such as the RAPIDO-protocol in patients with MRI-positive LLN and the role of PET-CT in diagnosing LLNM [32]. No current studies or recommendations regarding follow-up of these patients after CRT and resection without LLND exist. However, intensified follow-up might be justified.

This study suggests that current protocol, combining neoadjuvant therapy with selective LLND in case of therapy-resistant MRI-positive LLNM, is an applicable strategy in terms of both local and distant recurrence risks. Further data, possibly on a national level, to support this treatment strategy is needed.

## Supplementary Information

Below is the link to the electronic supplementary material.

Supplementary file1 (DOCX 13 KB)

## Data Availability

Data could be made available upon request to the authors.
