# Supplementary material for: Management, treatment and prognostic significance of lateral lymph node metastases in rectal cancer—a regional cohort study
Source: Int J Colorectal Dis. 2021 Sep 6;36(12):2707–14. doi: 10.1007/s00384-021-04018-1 (PMC8589806; doi:10.1007/s00384-021-04018-1)
Supplement: Supplementary file 1 — Supplementary file1 (DOCX 13 KB) [file 384_2021_4018_MOESM1_ESM.docx]

Supplement 1

Suppl. 1 – Review protocol for MRI-evaluation. LN, Lymph node; MRF, mesorectal fascia.

Supplement 2

Suppl. 2 – Kaplan-Meier survival plot between MRI-negative (n=314) and MRI-positive (n=30) patients. LLN, lateral lymph node.

Supplement 3

Suppl. 3 – Figure over cM1-distribution in patients with MRI-negative and MRI-positive LLN. LN, Lymph node.

Supplement 4

Suppl. 4 – Flow chart of performed surgery in relation to tumour height and LLN-status. APR, Abdomino perineal resection; AR, Anterior resection.
